# Exploring trade prospects of Chhurpi and the present status of Chhurpi producers and exporters of Nepal

**DOI:** 10.1186/s42779-022-00165-0

**Published:** 2023-01-02

**Authors:** Rajendra Panta, Vinod Kumar Paswan, Prajasattak Kanetkar, Durga Shankar Bunkar, Hency Rose, Shiva Bakshi

**Affiliations:** Department of Dairy Science and Food Technology, Institute of Agricultural Sciences, Banaras Hindu University, Varanasi, 221005 India

**Keywords:** Chhurpi, Nepali Chhurpi, Ethnic fermented dairy product, Ethnic food, Dog chew

## Abstract

Chhurpi is the hardest cheese known in the world that is typically made in the mountain region of Nepal by coagulating milk with the help of coagulating agents and thereby partly draining the whey from the milk of Yaks, Chauris, Cows, and Buffaloes. Especially hard Chhurpi of Nepal is gaining popularity abroad as a dog food. However, the crosscutting issues of Chhurpi production and trade have remained largely unexplored. Therefore, to increase our understanding and add some information on the Chhurpi enterprise in Nepal, the present study was undertaken to investigate the current status of producers and exporters of Chhurpi and the crosscutting issues of the Chhurpi trade. The study was conducted during the month of July/August (2021) by preparing a different set of questionnaires for exporters and producers, for which five municipalities of Ilam were purposively selected for producers, while Kathmandu was selected for exporters as a study area. The study was performed during COVID-19 pandemic, so a survey was done via telephonic and electronic means at the Institute of Agricultural Sciences, Banaras Hindu University. After analysis of obtained data, results revealed that exporting companies are making annual average growth of 10–11% from the last five fiscal years resulting in an increment of export. With an increase in annual sales, exporters are earning 10–15% profit of sales which is surging each year. The percentage of annual export decreased recently in the fiscal year 2020/21 due to COVID-19 as demand was low so production was made lower. The study area was dominated by educated, middle-aged respondents who mostly were males. Brahmin and Chhetri were major ethnic groups among producers most of whom are involved in cooperatives. Most of the producers were earning up to NPR 5 lakhs (USD 3817) annually only from Chhurpi. Price variation among wholesalers and retailers was prevalent in the production area. Most producers have reared cattle and their major problem includes feed shortage, disease in animal and breed improvement. Production areas should be made Foot and Mouth Disease free zone by the government through specific laws to further increase export to new countries.

## Introduction

**Fig. 1:**
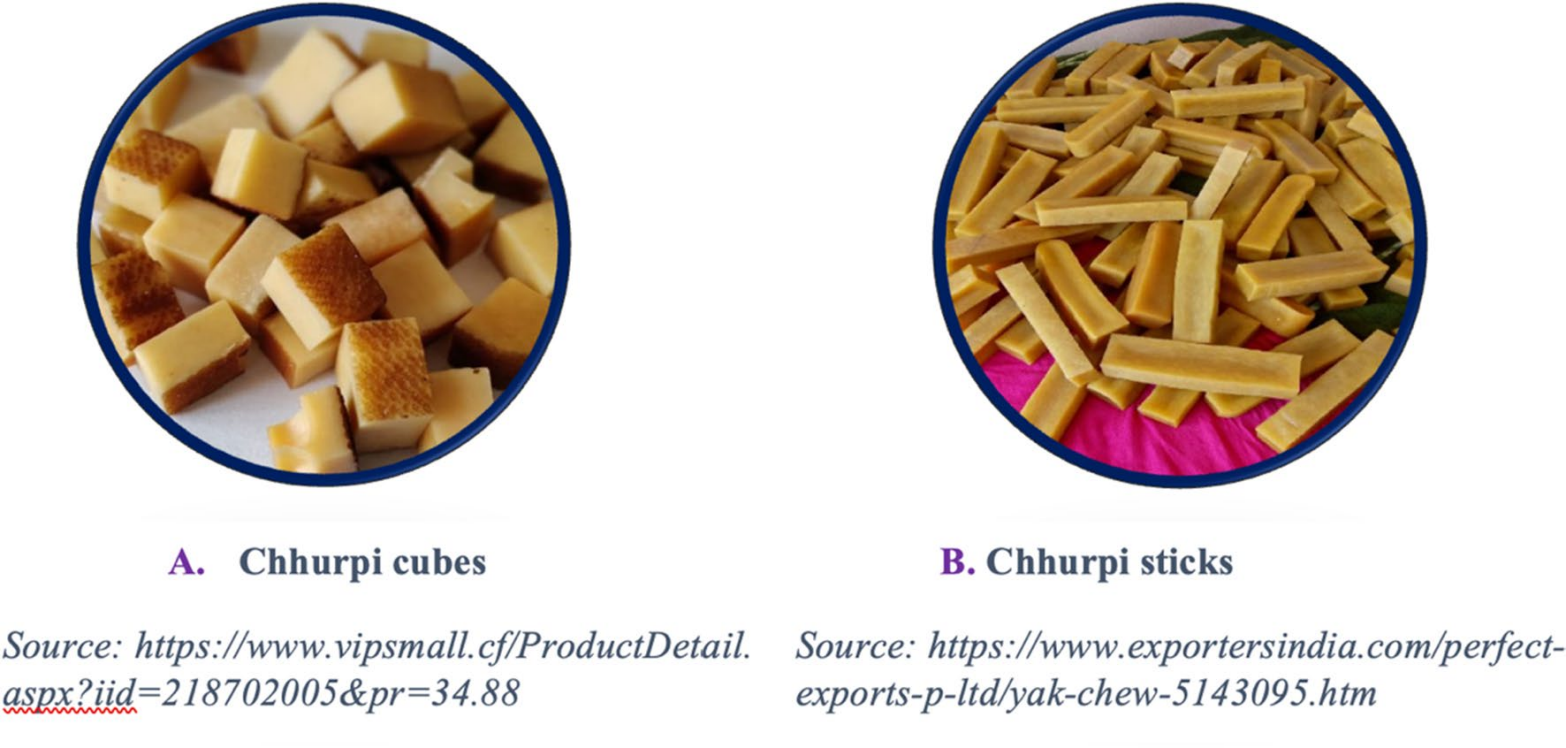
Chhurpi cubes (**A**) and Chhurpi sticks (**B**)

Chhurpi/Durkha is an indigenous fermented milk based pale-yellow cheese that is typically made within the mountain region of Nepal. The major fermented traditional foods of Nepal are Masyaura, Fulaura, Selroti, Sinki, Kinema, Khalpi, Mesu, Dahi, Mohi, Ghiu, Jandh, and Rakshi [1]. In addition to these, Chhurpi is one among them [2]. It is prepared by coagulating milk (whole or skimmed milk, partial or whole protein of the milk, buttermilk or any combination of the above materials) with the aid of calf rennet or any suitable coagulating agents (microbial or vegetable rennet, vinegar, lemon or backslopping) and thereby partly draining the whey [3]. Chhurpi is defined [2] as “a hardened cheese in the form of cubes and consumed by biting or chewing as betel nut”. A food is considered fermented when microorganisms have acted on its constituents to produce a product that is suitable for human consumption [4]. Nepalese Chhurpi has gained popularity in the international market. Chhurpi (defatted) is prepared typically in Nepal and exported worldwide as a dog chew in the form of cubes and sticks (Fig. 1).

Chhurpi is prepared from milk obtained from Naks (female Yaks), Cows, Buffaloes and Chauris. Chauris are cross-bred of Yak (*Bos grunniens*) with hill cattle (*Bos indicus*, Aule gai) and or Kirko (bull) with Nak [5]. Chauri grazes in alpine pastures (3000–4500 m) for about two months July–August and in the rest of the year, they remain at the lower altitudes of oak forests (2500–3000 m) [6]. In comparison with Chauris, yaks graze at a higher altitude. It is at 1100 m above sea level where the richest medicinal plants are found, while the greatest number of protected areas is located between 3000 and 3500 m above sea level [7]. Hence, Nepalese Chhurpi made from Yak and Chauris milk has more medicinal properties which account for its higher demand. Below that range, Chhurpi is prepared from mixtures of cow and buffalo milk.

The hard and soft type of Chhurpi is popular. Mainly Yak milk is preferred to make Chhurpi which produces the hard type whereas the soft variety of Chhurpi is prepared from cow milk. The soft type is used as a substitute for vegetables because of its richness in protein content. It is generally consumed with rice as it replaces curries and also as a filling material for momos. In Nepali cuisine, soft Chhurpi is used to make achar, and dumplings or eaten with edible ferns called sauneyningro in the local dialect. The achar is more or less like chutney prepared with finely sliced onion, and tomatoes with lots of fiery chillies called *dallekhorsani* and used as a side dish [8]. The hard variety can last up to 20 years or more if stored properly in the yak skin. The hard Chhurpi is used in efficiently removing plaque and tartar and keeps the gums strong. The flavour of Chhurpi can be best described as fresh and tangy. Chhurpi has interesting characteristics of chewiness and gumminess [9]. The final appearance, flavour and texture of finished Chhurpi depend on three parameters namely time, temperature, and acidity. Through the combination of these parameters, a shelf-stable Chhurpi can be prepared by utilizing Hurdled technology [10].

Traditional fermented foods have played an important part in human society since fermentation enhances the shelf life, texture, taste, aroma, and nutritional value of food [11]. Fermented foods and drinks have various health benefits. Fermentation transforms the agricultural substrates into new and often sensory-pleasing products [12], increment in shelf life and safety of products along with prevention of pathogenic contamination [13], reduction in anti-nutrients such as phytic acid that is bound to minerals in grains and via enzyme activity increasing the bioavailability of certain nutrients [14], enhancement in the nutrient availability including B-vitamins as well as antioxidant activity.

According to European Commission (2007), traditional foods are generally prepared and consumed by the local people of a particular region. Chhurpi (homemade cottage cheese) is also very popular among different ethnic groups in the Himalayan region of Darjeeling, Sikkim, Bhutan, Arunachal Pradesh, and Ladakh [15]. In these agroecological domains, people generally consume chhurpi as a masticator or add into curries [16, 17]. It is a 100% natural product that is free from gluten, grain, preservatives as well as artificial flavouring which is thought to be native to Tibetan cultural areas that spread to Nepal and India. Nepali Chhurpi is the hardest cheese in the world that is often called a Himalayan chewing gum [18].

Cheese is thought to have evolved before 8000 years around the Tigris and Euphrates rivers, which are located now in Iraq when people started domestication of plants and animals [19]. Nomadic tribes from Central Asia were the first to make cheese [20]. Over 1400 different kinds of cheese have already been recorded in the Centre for Dairy Research of the University of Wisconsin [21]. Chhurpi making history goes back to thousand years in the remote regions of Himalayas [22]. Nonetheless, the first Nepalese cheese industry was set up in 1953 when the government initiated the production of Yak cheese in the Langtang and Rasuwa districts of Nepal after getting support from the Food and Agriculture Organization (FAO). It has been reported that in the areas of Langtang and Ilam, several small-scale cheesemakers have begun production of Swiss Emmental and French Cantal types of cheeses [20]. At present, there are 15 small and medium-scale cheese industries registered with the Department of Food Technology and Quality Control, Government of Nepal [23].

There is a global market of Nepali Chhurpi. Mostly, this Chhurpi is found in the western market as a dog food since dog populations are higher there. According to a survey conducted among pet owners, the USA alone have an 89.7 million dog population in 2017 [24]. In the recent trend of marketing the product either in the national or international arena, the use of information and communications technology (ICT) has been a competitive tool to achieve the target [25] as the internet is believed to be vital in searching for information during food purchasing [26]. Using social networks and e-commerce in trading is famous worldwide. Any firm can achieve a secure and robust online position via the use of websites if informative, relational and transactional functions are effectively linked with the online commerce of ethnic commodities like Chhurpi. Many countries are adopting e-commerce as a better tool for marketing, particularly for their ethnic products. Major Indian portal sites are shifting towards e-commerce instead of depending on advertising revenue [27]. Also, the growth of e-commerce will increase exponentially in the upcoming years in the Indian market [28]. Similarly, the use of ICT has been a boon to a nation like Tunisia, which has increased its business network, and economic efficiency and improved the relationship marketing strategy in the olive oil sector [29]. Because of the recent significant growth in the Indian and other foreign markets, traders in Nepal have started to follow this trend as well. The country's growing e-commerce can help the Chhurpi trade.

Milk and milk products, including Chhurpi, have many health and nutritional benefits to their consumers, consisting of protein, fat and other minerals like calcium and phosphorus [30]. It is a rich in energy that is popularly known as an “energy tablet” among people in hilly areas. It also has a good amount of protein (60–63%) and carbohydrates (23–24%); and a low amount of fat (7–8%) [15]. Cheese is also a generous source of many vitamins like vitamin B6, vitamin B12, and folic acid [31] and these vitamins help to reduce the risk of atherosclerosis. It is reported that Chhurpi contains 2–4% moisture [32]. In addition, lactose is lower in cheese as bacterial action converts it to lactic acid during cheese making and fermentation. Therefore, it can be easily consumed by people with lactose intolerance [33]. The main aim of this study is to know the present status of Chhurpi producers and exporters in Nepal and to explore the prospects of trade of Chhurpi in this country. This will contribute to a better understanding of the dairy industry and add some information about it, especially about the hard Chhurpi, a traditional dairy product of Nepal, about which only a limited amount of research has been done so far (Table 1).

**Table 1:** Nutritional composition (%) of Hard Chhurpi

Attributes	Composition (%)
Total solids	89.77
Protein^a^	63.33
Fat^a^	7.20
Carbohydrate^a^	23.17
Ash^a^	6.30
Moisture	10.23

^a^These attributes are expressed in % dry matter/ total solid basis

Source: [15]

## Materials and methods

### Selection of study area

**Fig. 2:**
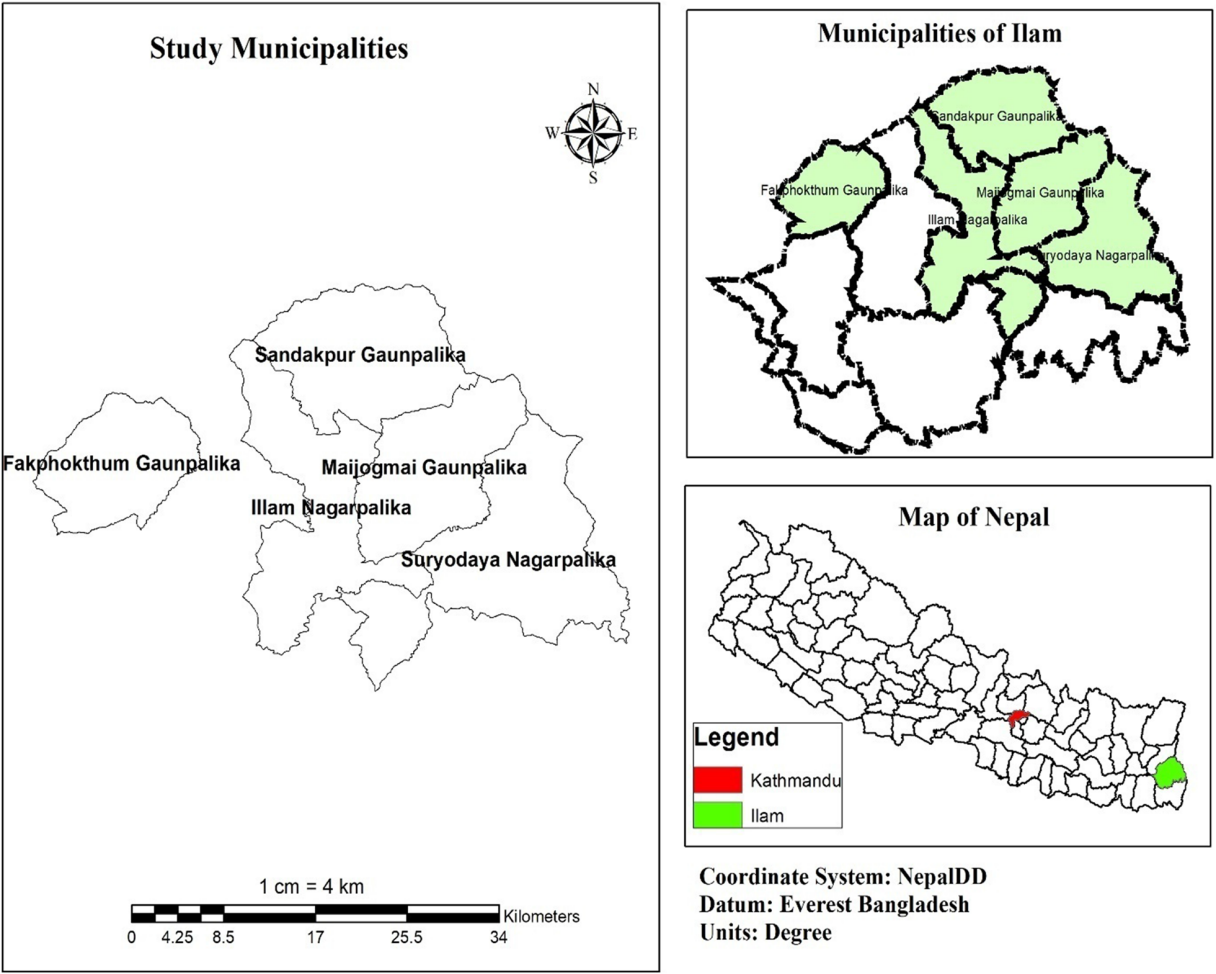
Study area of Chhurpi production and export in Nepal

To study the present status of Chhurpi producers and exporters a survey based on a pre-validated questionnaire was conducted via telephonic communication from 25th July to 11th August 2021. Electronic communication with certain exporting companies was conducted as well. The methodology employed for this study has already been effectively adopted earlier for the speedy acquisition of information [34]. Additionally, surveys adopting different means have often been chosen by various researchers as the most effective method of data collection for such studies [35]. Five municipalities in the Ilam district were chosen for the survey since major producers of chhurpi are located there, while for exporting company Kathmandu district were specially chosen. The production area under this study lies in the eastern part of Nepal while exporting area is in the central part of the country, which is also the capital city. Both the districts are well connected by road network. The distance between these two districts is 487 km. The study area of this survey is presented in Fig. 2.

### Selection of respondents

In order to conduct the study in a systematic and scientific manner and collect data from actual producers and exporters, a list of Chhurpi producers from Province 1, where producers are mainly found, was provided by the representative of the Ministry of Land Management and Agriculture Cooperatives of Nepal. Additionally, we found out about the producer’s organization (Dairy Farm), which gave us detailed information about the producers. From each study site, six producers were randomly selected. The main criteria for random selection were their involvement in producing Chhurpi (above five years). For the study, a total of 30 producers were examined. However, for exporters, a random selection was made from those who had been registered with the Office of the Company Registrar and had been exporting for more than five years. Nine such exporters were subsequently selected.

### Data collection, tabulation, and analysis

For collecting data, both primary and secondary sources were successfully utilized for the research study. As our primary source of information, we consulted a range of dairy-related actors and stakeholders, including milk producers, dairy cooperatives, milk marketers, dairy professionals as well as dairy industrialists and producers and exporters of Chhurpi. To collect the information from producers and exporters, two distinct questionnaires were prepared. Questionnaire about producers dealt with the socio-economic status of producers, land information, farming system they were involved in, livestock holdings, agriculture pattern, their involvement in cooperatives, their basic economic condition etc. The questionnaire for exporters was concerned with their business background, annual sales variation, annual income, major competitors, exporting countries, COVID-19 impact, government subsidies if received any, views of Nepalese consumers, suggestions to the government etc. Questionnaires were filled out in accordance to the information and data provided by the respondents. The questionnaire consists of both open and close-ended types of questions for both producers and exporters. The questionnaires were validated by presenting the list of questions to a small number of local respondents from similar target groups and corrections were made as per the feedback received from them. A total of 39 interviews, including both exporters and producers, were carried out.

The secondary source of information was reviewed from a number of local, national, and international studies, journal articles, case studies and conference presentations, both published and unpublished. The quantitative and qualitative methods were used to analyse data when it was entered into an Excel spreadsheet. Quantitative data were evaluated using descriptive statistics such as percentages and the use of graphics whereas qualitative data was presented by illustrations, simple tables, charts, graphs, and other pictorial forms.

### Limitation of study and future line of research

The study had major limitations because of the COVID-19 pandemic and its constrained time frame. Furthermore, it has been assumed that the information from the published annual report and telephonic communication is authentic and accurate.

## Results

This section is the outcome of the survey undertaken among Chhurpi producers and exporters of Nepal. The results are presented in three different sections dealing with relevant findings associated to Chhurpi exporters, Chhurpi producers and Marketing Channels of Chhurpi based on the data obtained from the primary and secondary sources of information collected during the study. The major key findings suggest that most of the producers are located in the eastern hills because it has a long history of such production trends while exporters are centred in Kathmandu valley as it has all sorts of a resources required by the exporting companies.

### Chhurpi exporters

#### Present status of exporters

In Nepal, Chhurpi exporters are mainly centralized in the capital city of the country (Kathmandu) because here only the international airport (Tribhuvan International Airport) is located till date. Also, all sorts of other amenities could be easily made available there. After 2003 the initiation of Chhurpi trading as a dog chew was started. According to the total data acquired from the exporters, on average they started their business in the past 6–7 years. Apart from Chhurpi, as per customer demand exporters are also exporting Chhurpi powder and Chhurpi Puff. Chhurpi is the only milk product that is exported to Europe and America (including all American states), and is fetching a premium price. Other countries for export include Canada, Hong Kong, Taiwan, Malaysia, Japan, Philippines. Most of them buy locally made Chhurpi from the farm located in the Ilam district while some exporters have their farms in Rasuwa and Langtang districts as well. Exporters stated that if the quality is not compromised then products can be exported across the globe as they are receiving good feedback from importers of their products. The quality of Nepalese Chhurpi is good as compared to other countries' products. It is common for Nepalese Chhurpi to develop moulds if it has to be transported for a long period of time.

#### COVID-19 impact and survival mechanism

Corona has negatively affected dairy activities despite the government's relaxation allowing most dairy operations to remain operational, even though the entire country was in lockdown. In the course of this COVID, almost all economic and public activities were shut down. The sale of liquid milk has decreased to a lower level along with other milk products. The stock of Skimmed Milk Powder (SMP), butter and cheese have been piled up in dairy industries resulting in blockage of capital which made dairies unable to pay the producers, staff, and banks. Therefore, to solve this situation dairy entrepreneurs were demanding soft loans/working capital to ease the regular operation of the dairies at a maximum of 3–4% for a period of at least 2 years.

As a result of the change in the economy, most exporters experienced a minor drop in their sales. It may be concluded that the importer's order decreases as the price of Chhurpi increases. To manage this critical period, the importer made the proper use of stock. To cope with this situation, exporters reduced the quantity of Chhurpi bought from producers. This condition does not last for long period but it disbalanced the trading status for a couple of months after the lockdown is initiated. Alongside, Chhurpi's cost has risen primarily due to the increase in feed supplements, as well as the higher transportation fees from the farm to Kathmandu as there was irregular transportation.

#### Major competitors in the market

Since the market of Chhurpi is growing consistently in the past 10 years, many new companies are being registered. But the main competition remains with those companies or firms that are operating in an unorganized way without being registered that are mainly private. Also, they can enter into space between organized companies and importers and thereby trying to attract the market towards them.

The survey found that the major players in Nepali Chhurpi export, have been in this business for more than 5 years with Himalayan Dog Chew being the top competitor in this scenario. Other companies that are boosting up Nepali economy with Chhurpi as major exporting commodity are Native Nepali Agro Supplies Pvt. Ltd., Omega Pet Foods Exports, International Himalayan Dairy Industries Pvt. Ltd., Zephyr International Pvt. Ltd., Continental Cashmere Pvt. Ltd., Unique Organics Pvt. Ltd. Kathmandu Nepal, Made in Nepal Pvt. Ltd., Fireball Trading Company Pvt. Ltd., Sudha Trading Pvt. Ltd., Nepa Dog Chew, Everest Dog Chew, Silver Lining, Godawari International, Naulo Venture, etc. Most of these companies are located in Kathmandu while some of them are operating from abroad as well as having production units in Nepal. Their major trading items include Dog treats, Dog chew, Dry Dog foods, Chhurpi powder, Puff, etc. A healthy competition between these competitors will likely increase the total exports, ultimately accelerating revenue collection.

#### Importer’s milk preference

Most of the country prefers Chhurpi made from Yak milk while some prefer that of Cow. The mixture of cow and buffalo milk is also subjected to preference in some countries and the milk of Chauri is also popular among importers. Among all these species, the Yak lives at a higher altitude and grazes in highland pastures that are full of medicinal and herbal plants. The quality of milk it produces is rich in medicinal and nutritional properties that is why it has more demand. While some found its milk product unpleasant as their dogs are already habituated with that of Cow milk. Other element responsible is the population of Yak/Nak/Chauris in the country with their numbers limited to 69,978, out of which the milking population is only 18,359 [36]. This is creating pressure on producers and also the grazing land shortage is an eminent problem.

#### Seasonal fluctuation in sales of Chhurpi

Although Nepal does not have a tradition of feeding dogs with Chhurpi, Nepali Chhurpi is gaining popularity outside Nepal as a chewing food for dogs. It is because the dog can be kept calm, quiet and under control, for 4–5 h while it is busy chewing. It is beneficial for dog health as well, being rich in surplus nutrition.

The market of Chhurpi remains almost the same throughout the year for most of the Nepalese exporters, even though some exporters reported a decrease in sales during the rainy season. But it was found that the sales within foreign market increase somehow during the festive seasons like Christmas, New year etc. as foreigners have a tradition to give a treat to their dogs in these seasons. People with no dogs also buy dog chew and offer it to their neighbours’ dogs which ultimately increases the sales of Nepalese Chhurpi.

#### Annual growth of Chhurpi export

**Fig. 3:**
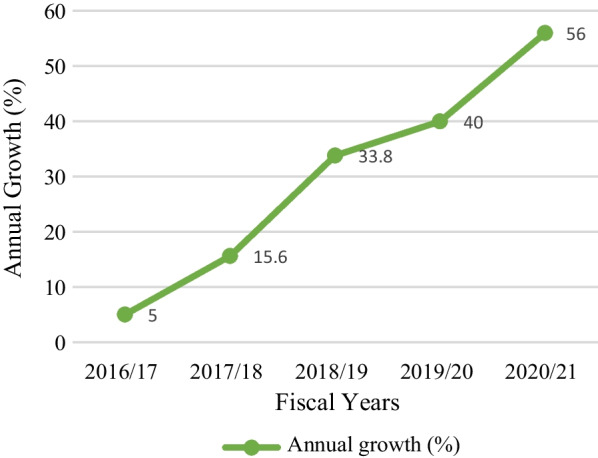
Annual growth in sales of Chhurpi exporter

Figure 3 is the representation of the increase in annual growth of sales in Chhurpi export in the last five fiscal years. Exporters who were having only 5% growth in 2016/17 increased their growth rate up to 56% while coming in 2020/21. In between these years also they have shown a continuous rise in their growth 15.6% in 2017/18 and 33.8% in 2018/19 while in 2019/20 exporters were having 40% growth.

This trend shows that growth in this sector is consistent and can always be made up going if proper care is taken in managing the market abroad. Since it is rapidly growing with an average growth rate of 10–11% annually the competition in this line is certain to increase in future.

#### Annual variation in Sales, Profit and Export of Nepali Chhurpi

**Fig. 4:**
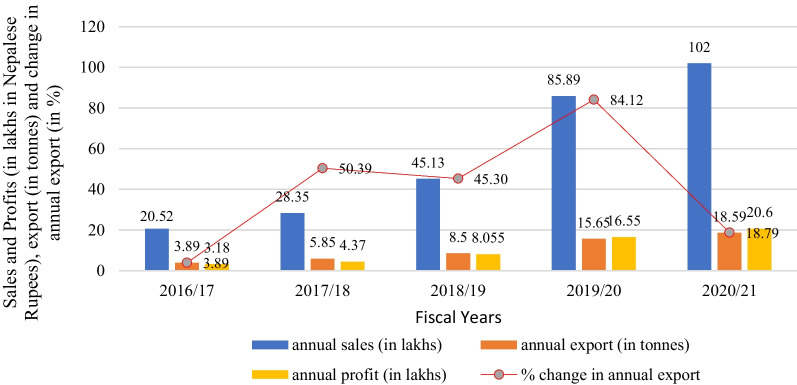
Annual variation in sales, profit, and export of Chhurpi in the last five fiscal years

The data associated with annual sales (in lakhs in Nepalese Rupees), annual profit (in lakhs in Nepalese Rupees), annual export (in tonnes) and percentage change in annual export of Nepali Chhurpi is depicted in Fig. 4. It can be noted that in the recent fiscal year (2020/21) all the three parameters have shown superiority in terms of annual sales (102 lakhs), annual profit (20.6 lakhs) and annual export (18.59 tonnes), while all these parameters remain at a lower level during 2016/17 being annual sales (20.52 lakhs), annual profit (3.18 lakhs) and annual export (3.89 tonnes).

In between these fiscal years, all three parameters have an increasing trend throughout the period while the maximum quantity exported was 15.65 tonnes in 2019/20 which was only 8.5 tonnes in the previous fiscal year. In the fiscal year 2019/20 the annual sale was 85.89 lakhs and the annual profit was 16.55 lakhs which remained at 45.13 and 8.055 respectively in 2018/19. There exists variation between each fiscal year in terms of percentage change in annual export. Annual export increased by 50.39% in 2017/18 which was only 3.89% in the previous fiscal year 2016/17. But in 2018/18 export percentage was reduced to 45.30% which again took a surge to 84.12% in 2019/20. During the last fiscal year 2020/21, only an 18.79% change was achieved in annual export. The reason behind this was the outbreak of COVID-19 which lowered the demand for Chhurpi. Similarly, to cope with this situation, export was reduced by reducing production as well.

### For Chhurpi producers

#### Present socio-economic status of Chhurpi Producers

**Fig. 5:**
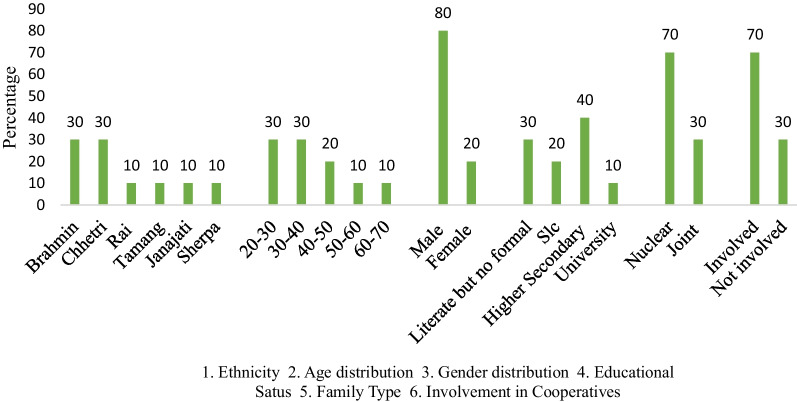
Socio-economic status of Chhurpi producers of the study area in Nepal

Nepal is a country where people with varying cultures, religions and languages are residing throughout the country. Ilam is in the easternmost part of Nepal and has a moderate type of ambience for dairy farming and is the reason why most Chhurpi producers are in this region. As for the ethnicity of the Chhurpi producers is concerned (Fig. 5), the maximum number of Chhurpi producers was found to be Brahmin (30%) and Chhetri (30%), while the rest of the other percentage share was divided equally with 10% each among Tamang, Rai, Janajati and Sherpa. Among the Chhurpi producers, it can be seen from the chart (Fig. 5) that the age of maximum Chhurpi producers lies between 20–30 years and 30–40 years, having 30% of producers in each category. From the observation, it is clear that youths are attracted to the production trend. 20% of producers were found to be between 40–50 years, while the least percentage of people were between 50–60 years and 60–70 years in which they share 10% in each category. Out of the total producers surveyed, more than three-fourth (80%) were male, whereas 20% were females.

In the context where dairy farmers and producers are not literate, Chhurpi-producing farmers have shown opposite results where the maximum number (40%) of producers are those with higher secondary education. 30% of producers know to read and write without formal education, which is followed by those with School Level Certificates (SLC; 20%). The least number (10%) is of those who are university graduates. The maximum and major proportion (70%) of Chhurpi producers have a nuclear type of family while the remaining 30% of producers consist of a joint one. The maximum number (70%) of Chhurpi producers are involved in cooperatives, while only 30% are not involved with cooperatives. In the course of our survey, we noticed that producers who do not currently belong to cooperatives were also interested in joining them in the near future. Those who were already members of cooperatives took membership 5–6 years ago.

#### Income from Chhurpi production and marketing

**Fig. 6:**
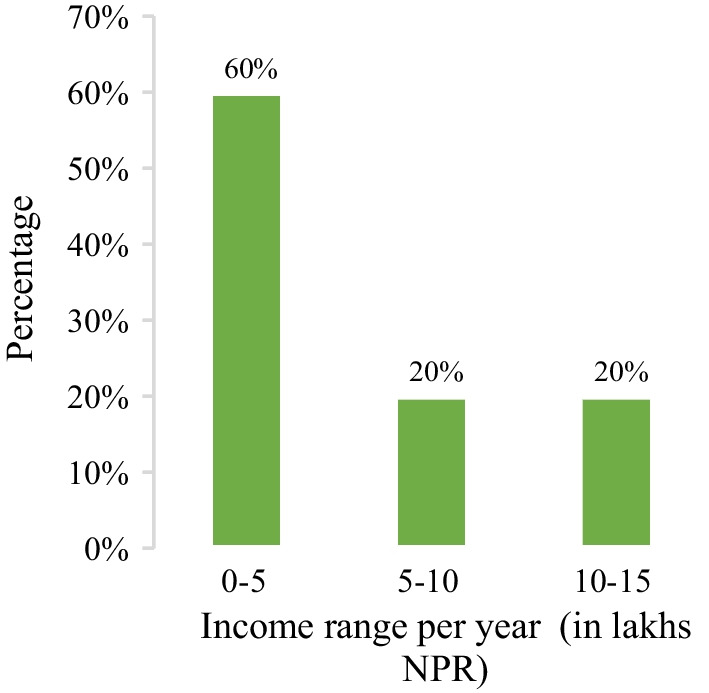
Income of Chhurpi producers. 1 lakh = 100,000; NPR Nepalese Rupee (1 USD =  ~ 131 NPR)

Chhurpi has been taken as a major exportable commodity in Ilam for years ago. Many new enterprises minded individuals are involved in this business to explore its opportunities. The income range is classified into three categories; 0–5, 5–10 and 10–15 lakhs Nepalese Rupees(NPR) per year (1 USD =  ~ 131 NPR). It can be noted that the highest percentage (60%) are earning income between 0–5 lakhsNPR per year while those earning between 5–10 and 10–15 lakhs NPRper year are 20% in each category (Fig. 6).

#### Annual Price variation of Chhurpi among seller (wholesaler and retailer)

**Fig.7:**
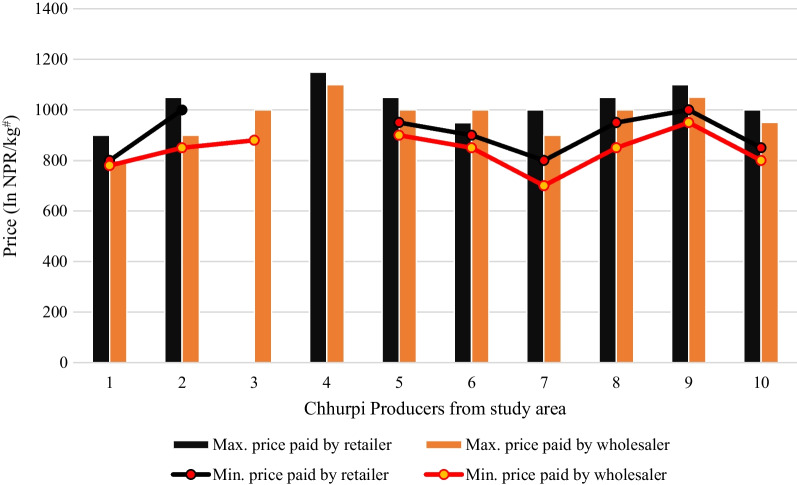
Variation in price (NPR/kg) of Chhurpi. ^#^NPR Nepalese Rupee (1 USD =  ~ 131 NPR)

The price (NPR/kg) of Chhurpi is mostly fixed across the year but it can vary if the producer sells it to others. The annual variation of selling price among each producer to wholesaler and retailer has been presented in Fig. 7. It can be noted that there is no result of retailing price in the case of producer number 3, because this producer only sells Chhurpi to the wholesaler at a maximum price of NPR 1000 (USD 7.63) and minimum at NPR 880(USD 6.72).

It is evident that the maximum price paid by the wholesaler and retailer was NPR 1100(USD 8.40) and NPR1150 (USD 8.78) respectively for producer number 4 but there is no minimum price in this case because the producer has fixed the price of Chhurpi in yearly basis (Fig. 6). A producer that has received a minimum price from both the wholesaler and retailer is producer number 7. They have received NPR 800(USD 6.11) from the retailer and NPR 700(USD 5.34) from the wholesaler respectively. All other remaining producers have got a price variation between the highest and lowest price received from the wholesaler and retailer. Furthermore, it was noted during the survey that this change in annual price variation is due to producers earning more profit, selling their product to a new exporter who is paying little more, lowering their image among previous buyers.

#### Crosscutting issues of Chhurpi producers

##### Sources of animal feed

**Fig. 8:**
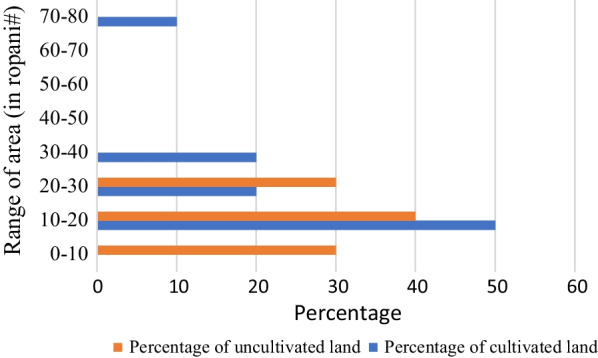
Annual growth in sales of Chhurpi exporter

Maximum cultivated land area (50%) was in the range of 10–20 ropani (0.51–1.02 hectare) whereas minimum cultivated land area (20%) was in the range of 30–40 ropani (1.53–2.03 hectare) (Fig. 8). Maximum uncultivated land (40%) was in the range that was similar to maximum cultivated while minimum uncultivated land (10%) was found in the range of 70–80 ropani(3.56–4.07 hectare). Chhurpi producers were found to grow fodder and forage on their land, while concentrate was bought from nearby agro vets and agriculture inputs suppliers.

The available land area was divided into a section of fodder production and crop production. The crop residues like wheat and rice straw were also used in feeding. The land area that was allocated for fodder plantation included plants like Amriso (*Thysanolaena latifolia*), Khanayo (*Ficus semicordata*), Nemaro (*Ficus auriculata*), Dudhilo (*Ficus neriifolia*), Kutmero (*Litsea polyantha*), Kimbu (*Morus alba*), Gogan (*Saurauianapaulensis*), and Bar (*Ficus benghalensis*), while Napier (*Pennisetum purpureum*) and Alfa-alfa (*Medicago sativa*) were planted as a forage crop. Furthermore, from the survey, it was found that almost all producers have their own land for cultivation, most of which is upland and have good provision of irrigation. Very few landowners struggle with rain-fed issues.

##### Selection of dairy animals among Chhurpi producers

The selection of animals plays a crucial role in the success of any dairy farm. Improved cow and buffalo breeds are the potent producers of milk while local animals are low milk yielders. Producers were found to rear improved breeds of cattle (Jersey and Holstein) and buffaloes (Murrah), while local breeds are not much popular in that area in terms of Chhurpi production.

It has been observed that maximum producers have a preference for cattle (40%). The percentage of producer-rearing buffalo only is 0% while those having both animals on their farm were 30%. The remaining 30% are the producers that do not own any animals but collect the milk from MPC (Milk Producers Cooperatives) to produce Chhurpi. The land holdings of such producers were found nil therefore they do not cultivate any fodder and forage species. It is believed that mixed milk from both cattle and buffaloes is the best for good-quality Chhurpi production. Milk only from buffalo has problems like breakage and uneven surface after it gets solidified. In order to overcome this problem, buffalo milk is mixed with cattle or yak milk. After milk (cattle and buffalo) gets homogeneously mixed, it results in Chhurpi with a smooth and uniform surface that is mostly preferred by consumers.

##### Milk collected or produced, and milk used for Chhurpi making

The majority of Chhurpi producers are those with less capacity of milk collection or production because they either have a limited number of farm animals or purchase a low amount of milk from cooperatives.

**Fig. 9:**
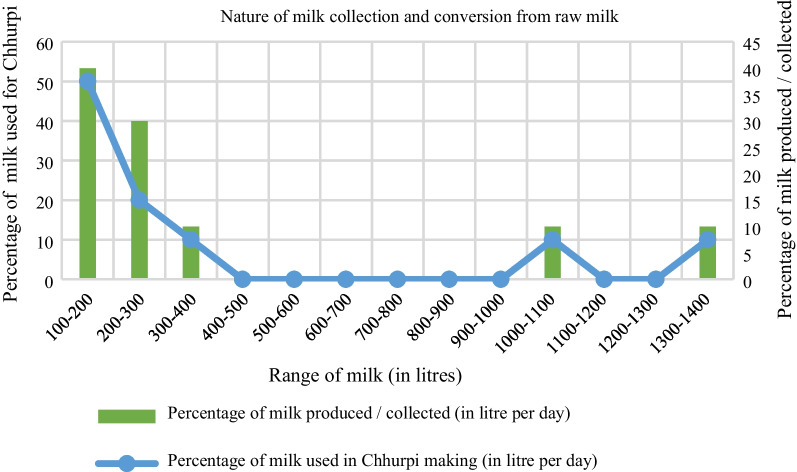
Cultivated and uncultivated land area among Chhurpi producers. #1 hectare =  ~ 19.65 ropani

It is evident that the maximum collection or production of milk (50%) lies in the range of 100–200 L while the minimum (10%) lies in the range of 300–400 L which share the same.

percentage as that of 1000–1100 and 1300–1400 L (Fig. 9). Highest range of milk that is used for Chhurpi making is 100–200 L which have half percentage share of total milk used while the minimum percentage of milk (10%) used in the conversion is 300–400 L which are also similar for 1000–1100 as well as 1300–1400 L.

#### Problems faced by Chhurpi producers

##### Higher costs of milk

Milk producers have to buy agricultural inputs at high costs and there is an absence of a mechanism to regulate farmers' cost of milk production according to their incurred cost leading towards an abrupt rise in the price of milk for Chhurpi producers. Another reason for the price hike of milk is the shortage of the required labourer’s number in the farm.

##### Feed shortage

During the monsoon period there is excess green forage and fodder available but after the monsoon remaining six months of winter and spring there starts a shortage of feed and fodder. In this situation animals are fed with concentrates that are too costly. This does not meet the requirement of animals which increases cost without an increase in production. This leads to a scarcity of milk, especially in the lean season. The lean period is known as risk season because the demand for production cannot be reached.

##### Storage and transportation

One of the major problems with Chhurpi producers was the storage of Chhurpi as it is hygroscopic and can easily absorb moisture. According to them, the maximum price they acquire per kg of Chhurpi (NPR 1100; USD 8.40) gets reduced to NPR200(USD 1.53) if Chhurpi gets moistened. So, transportation and storage of Chhurpi depend upon the types of Chhurpi (hard and soft). Hard Chhurpi should be stored at 8–10 °C. Such type of Chhurpi during the winter season can be transported without chilling (if the maximum temperature is 20–21 °C) for 1–2 h, and if the maximum temperature is lower than 15 °C the entire transportation does not require any chilling.

##### Animal health

The health of the herd is vital for producing the optimum level of milk. When it comes to the major infectious diseases in animals, Foot and Mouth Disease (FMD), Haemorrhagic Septicaemia (HS)and Black Quarter (BQ) are most common in Nepal. Henceforth, the government launched a nationwide FMD control project focusing on the vaccination of animals in pockets area of the dairy sector, Ilam is one of them. A periodic deworming and awareness campaign has been implemented for the same in the country.

##### Breed improvement

To meet the demand in the lean season and to maintain a regular flow of products to the market regular supply of milk to producers is crucial. For this local animals are not best suited and improved breeds of animals like Murrah in buffalo and Jersey and Holstein-Friesen in the cow are the option for boosting milk production. Good-quality semen of superior bulls should be used for Artificial Insemination (AI) in cows and buffaloes.

##### Technical knowledge

Chhurpi producers do not have adequate knowledge regarding different technical aspects of production. Milk should only be marketed after its technicality has been assured by the experts because Chhurpi producers have faced clotting problems in milk while boiling. There should be a proper understanding of demand and amount of milk production to overcome the problems of milk holidays. By boosting up technical manpower adequate technical support on vaccination, treatment of diseases, forage and fodder cultivation, ration formulation, preservation of fodder via silage making etc. should be taught to the farmers and producers.

#### Suggestion of Chhurpi producers and exporters to the government

##### Policy framework

It is the prime role of the government to enact a feasible policy regarding the market of Chhurpi. To increase the production of milk and milk products, small farmers should be given topmost priority in policy because the dairy sector of Nepal has a large percentage of small milk producers. The dairy cooperative model of milk collection should be framed and made functional in milk-producing sites. The business environment can only be created when government work on framing effective plans and policies for the overall development of the Chhurpi market.

##### Government subsidy

Governmental subsidies are essential in encouraging entrepreneurs to start their businesses. Chhurpi producers are urging the government to provide subsidies only based on the performance of producers. To boost the production of Chhurpi, the government should also offer subsidies for equipment so that it eases the preparation process. Subsidies are mostly provided to export-oriented industries, prioritized industries, as well as industries located in the least developed, undeveloped, and underdeveloped regions of Nepal. A technological subsidy can aid in tremendous ways to surge production.

Government should be proactive in providing training to the milk-producing farmers and the Chhurpi producers to educate them with technical information about Chhurpi. This should provide remunerative and subsidies for the inputs required for production. A high bank interest rate of around 16% is working as a prime hurdle in the growth of Small and Medium Sized Enterprises (SMEs) in Nepal. The government should direct the banking sector to allow such businesses to work by lending a nominal interest rate. Taxation should be subsidized for entrepreneurs as a result business environment would be created that ultimately would make a win–win situation for both the exporter and the country.

##### Foot and Mouth Disease (FMD) free zone

It is suggested to the government by both the producers and exporters of Chhurpi, to create FMD free zone in Ilam and other Chhurpi-producing districts. It can be done by adopting Sanitary and Phytosanitary (SPS) measures to eliminate health problems in animals thereby preventing them from major health diseases. After that, a clean certificate can be generated to the area that is specifically free from FMD or any other disease. The lack of a certificate prevents exporters and producers from entering the Australian market as Australia does not import milk and milk products from countries with animal health problems.

### Marketing Channels of Chhurpi

Most of the Chhurpi produced in the study area is marketed through varied channels at different prices. The channels that were prevalent in the study area for Chhurpi marketing include the following:

- Channel I: Producers → Exporters
  This channel is characterized by the direct selling of Chhurpi to exporters by producers. Generally, a contract is made between producers and exporters for a certain period and the price of Chhurpi is fixed without any scientific basis in mutual understanding between producers and exporters. There are also some exporters with production facilities in the study area.
- Channel II: Producers → Collectors → Exporters
  In this channel producers and exporters are connected by collectors. Here collectors may be MPC, Dairies, etc., that purchase the products from producers in bulk at a reasonable price and sell to the exporters making some profit in between.
- Channel III: Producers → Middlemen → Exporters
  The channel involves the middlemen as connection between producers and exporters. Middlemen purchase Chhurpi from producers and sell it to exporters. Middlemen are generally from the place of production, being local they have a good relationship with the producers and thus can collect the product easily. Most middlemen remain in the Terai area and develop a market link with other personnel to build up the business. Birtamode of Jhapa district is the place where many such middlemen of Chhurpi reside.
- Channel IV: Producers → Wholesalers / Retailers → Exporters
  In this channel, producers firstly sell their products to wholesalers in large quantities. Wholesalers collect enough Chhurpi to store at their store and then sell that product to exporters mainly in Kathmandu. Occasionally, producers have also been found selling their products to local retailers when they do not have surplus quantities.

## Discussion

The result obtained from the socio-economic status of chhurpi producers revealed that the maximum producers were Brahmin and Chhetri, while the rest of the ethnic groups were limited to a few. This result is consistent with the report of the Census 2011 conducted in Nepal, where the major caste was reported to be Chhetri (16.6%), followed by Brahmin-Hill (12.2%). Since our study site was on the mid-hills of eastern Nepal, this census status supports our findings better. Also, a similar result was obtained in a case study of the socio-economic condition of dairy farmers conducted in Lamahi, Dang [37].

The age distribution of producers revealed that the maximum number of people involved in Chhurpi production was in between 20–30 and 30–40 years of age. This result concurs with the findings of Paudel [37] and Nepali [38], where the maximum working youth population of Nepal was reported to lie between 25–45 years of age group. This research finding has a minimum percentage of producers above 60, which is against the finding of some researchers from Bahrain [39]. Moreover, Nepali reported that male farmers were more prevalent than female farmers when it came to raising livestock [38]. Our results comply with the findings of this researcher. In the end it shows male dominated production, whereas women aided in other works like fodder collection and feeding animals.

The educational status of Chhurpi producers in the current study was partially in line with Nepali [38]; as they reported that the percentage of farmers having higher secondary education was the second highest, while in our study, such farmers ranked first. These graduates entered this area since their family business used to be in the production industry and they had technical knowledge about it. Additionally, youth like them have broader links in the market allowing them to sustainably compete. Nonetheless, family structure of producers found in our study strongly coincides with the findings of Nepali, i.e. the majority of the producers belong to nuclear families [38] this might be due to the movement of the people from hilly areas to Terai in search of a better opportunity, to find a job, or education etc.

There are more producers who are active part of cooperatives, this is consistent with the findings of Neupaneet al. [40]. It may be majorly due to the facilities offered to members of the dairy cooperative. Some these facilities are easy availability of feed, agricultural inputs, and dissemination of technical knowledge. Tatlonghariet al. [41] have also derived that well-established cooperatives would facilitate access to information, and the transfer of innovation and communication, leading to an increment in its members. Similarly, Niroula (2003) has also agreed that the role played by cooperatives in socio-economic impact is significantly positive [42]. Therefore, even those who were not involved in cooperatives while surveying were also planning to be a member in the coming days.

It was determined that those earning 0–5 lakhs NPR(1lakh is 100,000) per year are doing other agricultural jobs, so they are not making much money out of it, and this group mostly includes producers collecting milk in less quantity. On the contrary, producers earning 10–15 lakhs NPR were found to collect more than 500 L of milk daily, resulting in such income. This finding of our study is further supported by the research carried out by Chaudhary and Upadhyaya (2013), whom reported that the earnings of a family involved in the dairy sector were higher than those involved in multisector [43]. Similar conclusions were made by Uotila& Dhanapala (1994), regarding income from the dairy sector, where the role played by dairy cooperatives was the critical factor for improving the living standard of rural producers [44]. Many farmers raised cattle rather than buffalo because cattle had a larger yield potential. This result is consistent with other studies findings that supported employing cattle to reduce poverty [45].

The main issues that Nepalese Chhurpi producers deal with are consistent with the findings of Joshi and Bahadur (2015) [46]. Similar problems related to animal health, fodder shortage, and technical expertise were faced by Srilankan dairy farmers [47]. Guadu and Abebaw (2016) outlined similar challenges among dairy farmers in Ethiopia, where farmers faced problems regarding poor breeding knowledge, inadequate feed resources and poor pasture development, reproductive and health problems, institutional challenges, and limited availability of credit to farmers [48]. Researchers from the National Cattle Research Program in Nepal identified related concerns with cattle farming in their study [49] and the exporters and producers suggested similar reform action from the government.

The status of Nepalese Chhurpi exporters is in ameliorating condition. Their number is progressively increasing in Nepal. Our collected data suggest that the market of Chhurpi is ever-expanding, and exporters forecast that the future market will be more concentrated in America. The reason behind this is due to the large dog population (89.7 million) in America [24], and dog owners spent 442 US dollars on pet food per year in 2020. Many traders are into this business for a long time. Our conclusions about the main competitors of this company are consistent with the World Bank Report [50]. Exporters have their major production unit in the eastern high hills. The result obtained from our analysis is supported by an article on Onlinekhabar [51], which delineated that the Ilam district is the prominent hard cheese-producing hub in the country. The majority of the numerous products made from chhurpi are exported to markets in the western world [52].

The aftermath left by COVID-19 was unbearable. A nationwide lockdown by the Government of Nepal was imposed from 24 March 2020 to 21 July 2020, prohibiting domestic and international travel, closure of the borders and non-essential services. Even after the complete lockdown, exports were affected by the partial lockdown which extended till late 2021 in different parts of the country. Nevertheless, exporters survived the situation of COVID-19. This finding concurs with major publishers [53]. The annual export growth between 2019–20 and 2020–21 decreased unexpectedly. The primary cause for this was a break in the value chain of milk due to the lockdown, when farmers were compelled to dump milk and veggies as a result of the closure of processing companies, which led to a price hike and a lack of supplies [54]. While exporters were facing critical conditions and demanding soft loans, the government launched an economic support package including deferred payment on tax, discount on interest rates and utility payments that cost approximately 5% of the GDP [55].

Major exporters reported minor changes in the sale of products as per our findings. The main reason behind this is the rapid acceleration in mobile and digital technology use amid the pandemic [55]. Some of them who relied on digital platforms reported increased sales during the pandemic [56]. During the lockdown, internet users have increased by 35%, which has boosted local markets and increased participation in social media and e-commerce [57]. This new opportunity was thought to increase the growth of the business from the viewpoint of entrepreneurs [58], as the emergence of digital technologies has offered opportunities among small and medium enterprises to enhance their reach, cost efficiency and competitiveness [59]. After experiencing such unexpected health havoc and seeing the prospects of online trading, the Nepal government is committed to utilizing e-commerce as a vehicle for development [60]. COVID-19 left heterogeneous impacts on Nepalese industries as well. The trend of import and export was altered too [61]. According to our research, Chhurpi's gathering from many manufacturing sectors is essential for it to remain competitive. The role that the collector plays in this scenario is commendable. This marketing channel is supported by Msaddak et al. [62], who have reported that the collector plays a vital role in the case of dairy transactions.

## Conclusion

Chhurpi has been an underutilized dairy product in Nepal, but it is gradually becoming more and more popular among dairy farmers. Promising a virgin and untapped export market as pet food, this indigenous Nepali dairy product has a high potential for income generation for farmers and traders. However, producers are facing problems like higher milk costs, lack of storage and transportation facilities, constraints in breed improvement and animal health, limited technical knowledge about the production process and lack of government subsidies. Management and policy interventions for alleviation of all these constraints, creation of FMD free zone in production hubs, financial assistance to farmers and entrepreneurs in the form of government subsidies, quality improvement of Chhurpi and trading via e-commerce are likely to boost foreign market sales of this product further.

## Data Availability

The data and materials were obtained based on survey conducted for the study. The whole data for this survey is not publicly available. However, many portions of data obtained from survey are incorporated in body of this article.
